# Factors to Effective Clinical Experience, Willingness to pursue Career in Rural Health Facilities among Nursing Students on Clinical Placement in Southeast Nigeria and Rural Development

**DOI:** 10.17533/udea.iee.v42n2e09

**Published:** 2024-07-06

**Authors:** George, O. Abah, Samuel, O. Okafor, Orkuma Anyoko-Shaba, Onyedikachi C. Nnamchi, Ekaette O. Ọkop, Akindele Ogunleye

**Affiliations:** 1 Senior Lecturer. Philosophy Department, University of Nigeria, Nsukka. Email: george.aba@unn.edu.ng University of Nigeria, Nsukka Philosophy Department University of Nigeria Nsukka Nigeria george.aba@unn.edu.ng; 2 Ph.D. student and research consultant. Department of Sociology/Anthropology, University of Nigeria, Nsukka. Email: samuelokey200@gmail.com. Corresponding author University of Nigeria, Nsukka Department of Sociology/Anthropology University of Nigeria Nsukka Nigeria samuelokey200@gmail.com; 3 Lecturer. School of General Studies, University of Nigeria, Nsukka. Email: okuma.anyoko-shaba@unn.edu.ng University of Nigeria, Nsukka School of General Studies University of Nigeria Nsukka Nigeria okuma.anyoko-shaba@unn.edu.ng; 4 Lecturer. Department of Psychology, University of Nigeria, Nsukka. Email onyedikachi.nnamchi@unn.edu.ng University of Nigeria, Nsukka Department of Psychology University of Nigeria Nsukka Nigeria onyedikachi.nnamchi@unn.edu.ng; 5 Lecturer. Department of Adult Education and extra Moral Studies, University of Nigeria, Nsukka. Email: ekaette.okop@unn.edu.ng University of Nigeria, Nsukka Nigeria; 6 Consultant. EI Paso Educational Leadership and Foundations, University of Texas, USA. Email: aogundele@miners.utep.edu University of Texas USA

**Keywords:** rural health services, clinical competence, public health, students, nursing, Nigeria, servicios de salud rural, competencia clínica, salud pública, estudiantes de enfermería, Nigeria, serviços de saúde rural, competência clínica, saúde pública: estudantes de enfermagem, Nigeria

## Abstract

**Objective.:**

To describe the Factors to Effective Clinical Experience and Willingness to pursue Career in Rural Health Facilities among Nursing Students on Clinical Placement in southeast Nigeria.

**Methods.:**

The study was conducted among 48 rural health centres and general hospitals with 528 respondents from different higher institutions of learning serving in these health facilities for their clinical experience. The study applied survey design and utilized questionnaire instrument for data collection.

**Results.:**

Majority of the students (60%) agreed that their school lacked functional practical demonstration laboratory for students’ clinical practice, 66.7% agreed that their school lab lacked large space for all the students to observe what is being taught, 79.9% that their school lab lacked enough equipment that can enable many students to practice procedures; majority of the students (79.9%) answered that the hospitals where they are on clinical placement lacked enough equipment needed for the students on each shift of practice, 59.9% agreed that student/client ratio in each ward during clinical experience periods was not enough for students' practice under supervision, while 73.3% indicated that their school lacked library with current nursing texts for references. Personal, socioeconomic and institutional factors explain the 76% of the variance of effective clinical experience and the 52% of the variance of the willingness to work in rural health facilities in the future if offered employment.

**Conclusion.:**

The factors surrounding effective clinical experience in rural healthcare facilities in southeastern Nigeria are unfavorable and could discourage future nurses from working there. It is necessary to implement strategies to improve the management of these centers in order to promote the perspective of improving sustainable rural health in this region.

## Resumen

**Objetivo.:**

Describir la experiencia clínica efectiva entre los estudiantes de enfermería en prácticas clínicas en las instalaciones sanitarias rurales en el sudeste de Nigeria.

**Métodos.:**

Este estudio descriptivo se llevó a cabo entre 48 centros de salud rurales y hospitales generales con 528 encuestados de diferentes instituciones de enseñanza superior que prestaban servicio en estas instalaciones sanitarias para su experiencia clínica.

**Resultados.:**

60% de los estudiantes comentaron que su centro carecía de un laboratorio de simulación para las prácticas clínicas de los estudiantes, el 66.7% indicó que el laboratorio de su centro carecía de un espacio para que todos los estudiantes pudieran observar lo que se enseñaba. Un 79.9% indicó que el laboratorio de su centro no disponía de equipos suficientes para practicar los procedimientos, y otro porcentaje igual (79.9%) manifestaron que los hospitales carecían del equipo necesario para realizar adecuadamente las prácticas clínicas. El 59.9% indicaron que la razón de estudiantes por paciente en cada sala durante los periodos de experiencia clínica era insuficiente para que los estudiantes realizaran prácticas bajo supervisión, y el 73.3% indicaron que los recursos de la biblioteca en textos de enfermería eran insuficientes para sus necesidades. Los factores personales, socioeconómicos e institucionales explican el 76% de la varianza en la experiencia clínica efectiva y el 52% en la disposición a trabajar en centros sanitarios rurales en el futuro.

**Conclusión.:**

Los factores que rodean la experiencia clínica efectiva en los centros sanitarios rurales del sudeste de Nigeria son desfavorables y podrían desanimar a los futuros enfermeros a trabajar en ellos. Es necesario implementar estrategias de mejoramiento de la gestión de estos centros con el fin de impulsar la perspectiva de mejorar la salud rural sostenible en esta región.

## Resumo

**Objetivo.:**

Descrever a experiência clínica eficaz entre estudantes de enfermagem em estágios clínicos em unidades de saúde rurais no sudeste da Nigéria (África).

**Métodos.:**

Este estudo descritivo foi realizado em 48 centros de saúde rurais e hospitais gerais com 528 entrevistados de diferentes instituições de ensino superior que atendem essas unidades de saúde pela sua experiência clínica.

**Resultados.:**

60% dos alunos comentaram que seu centro não possuía laboratório de simulação para as práticas clínicas dos alunos, 66.7% indicaram que o laboratório de seu centro carecia de espaço para que todos os alunos pudessem observar o que estava sendo ensinado. 79.9% indicaram que o laboratório do seu centro não possuía equipamentos suficientes para a realização dos procedimentos e outro percentual igual (79.9%) afirmou que os hospitais não possuíam os equipamentos necessários para a realização adequada das práticas clínicas. 59.9% indicaram que a proporção de estudantes por pacientes em cada sala durante os períodos de experiência clínica era insuficiente para que os estudantes realizassem as práticas sob supervisão e 73.3% indicaram que os recursos da biblioteca em textos de enfermagem eram insuficientes para suas necessidades. Fatores pessoais, socioeconómicos e institucionais explicam 76% da variação na experiência clínica efetiva e 52% na vontade de trabalhar em centros de saúde rurais no futuro.

**Conclusão.:**

Os fatores que rodeiam a experiência clínica eficaz em unidades de saúde rurais no sudeste da Nigéria são desfavoráveis ​​e podem desencorajar futuros enfermeiros de trabalhar lá. É necessário implementar estratégias para melhorar a gestão destes centros, a fim de promover a perspectiva de melhorar a saúde rural sustentável nesta região.

## Introduction

Health institutions across the world have become the centre of attraction in the recent history owing to their pertinence to the overall human survival anywhere in the world. While the developed nations have improved in the management of their health institutions and infrastructures with more innovative approaches, in the developing nations such as in sub-Saharan Africa, the situation is still struggling with a number of challenges mostly in the area of manpower.^1^^,^^2^Nurses are the pillar of the professional health workers designed theoretically and practical to sustain the health facilities in their capacities in collaboration with other health professionals and this finds its reality in the availability of functioning health facilities and management. Among other things, the nursing students through series of assignments and engagements in the institutions of trainings are prepared to competently meet the preventive and curative health needs of the population in the hospital and other settings after their trainings; these included the clinical placement and other health and hospital management-based programs during the period of their training in the institutions of higher learning.^3^^,^^4^ While these placements and other categories of engagements are designed to make these students competent and familiar with hospital settings towards their post-training service engagement, the engagement at this stage of their training equally contributes to their ability to contribute to the overall efficiency of the public health institutions as they graduate to continue their career in the hospitals and allied institutions.^5^^,^^6^ Consequently, the level of preparation and assimilation of the clinical and management knowledge at this stage among the students, become the bedrock for the preparation of another generation of nurses to sustain the hospitals and the health institution as a whole owing to the fact that, when these students graduate and get employed in the hospitals and allied institutions, they over time become the replacement of the retiring nurses and health management officials phasing out of the system. 

Clinical experience is one of the direct engagements with the hospital and health institutional/infrastructural settings by the nursing students during their trainings in the institutions of higher learning. While this engagement is one of the core aspects of their training, it is also observed as commitment to their training with much workload compared to other class works, they do. This is in view of the fact that, clinical experience is designed for the translation of the ‘theoretical’ knowledge of medicine and health management into practical dealings with preventive and curative health needs of a given population.^7^^,^^8^ Across times and regions, clinical experience among nursing students has been regularised to technically prepare the nursing students in institutions of higher learning, the hospital and health management, which are the chief of their career base. However, while this as part of nursing program has been stabilized world over in terms of timing in the student’s program and patterns of implementation, there appears to be challenges with its effectiveness and impacts among the students and the overall health system in different parts of the world such as in the developing nations. These challenges hover around some factors such as institutional, socioeconomic factors as well as personal factors among the students engaging in clinical experience. 

Institutional factors according to the study by Shokria, Chitra and Manal,^9^Woo and Li^10^ includes but not limited to organisational settings, management organogram, intra and inter personal and group relationship within the hospital setting between the students and the hospital staff as well as the patients. From the studies by Gemuhay, Kalolo, Mirisho, Chipwaza and Nyangena,^11^ Jafarian-Amiri, Zabihi and Qalehsari,^12^ socioeconomic factors among the students such as family background, ability to cope with the financial and other demands of the training, marital status and other similar factors all together have their impact on the commitment and performance of the medical students during their clinical experience. Nevertheless, personal factors such as attitude to clinical placement, anxiety, lack of self-confidence, absenteeism and engagement with step by step mentorship and assignment by the preceptors during clinical experience have significant impacts on the ability of the students to successfully engage and complete their clinical experience.^13^^,^^14^

According to the studies by Alshammari *et al*.^15^ clinical experience among the nursing students in places around Middle East is fraught with such challenges as institutional framework; inter personal relationship with the preceptors, poor organizational arrangement framework of operation to accommodate the students as well as poor clinical facilities lacking basic instrument and equipment for learning in hospital setting. The study by Mbakaya *et al*.^16^ carried out in Malawi, showed that hostile environment, poor relationship with a qualified staff, absence of clinical supervisor and lack of teaching and learning resources affected the clinical experience among the nursing students. According to the study by Fooladi *et al*.^17^ in Australia, nursing students posted to the clinical facilities faced with the challenges of lack of preceptors and effective supervision due to the hospital protocols, which lacked specific arrangement for the students coming for clinical experience in the organogram. Also, another study in Australia revealed that due to the nursing students who come for clinical experience in most cases lacked readily available skill to be used in hospital services, the preceptors and hospital management treat them as liabilities and as such gave them poor attention in supervision and assignment of duties.^18^ This according to the study further demoralised the students and affected their participating in clinical placement. 

The study by Salim *et al*.^19^ showed the impacts of marital status on the nursing students participating in clinical experience in Doha Qatar. According to the study, married students are appeared to be wearied in the clinical placement activities and showed little or no aptitude for the learning processes in clinical placement in the hospital setting. From the findings of the study by Trede *et al*.,^20^ clinical experience among the students in Canada appeared to be complicated with perceived unfriendly preceptors who gave little or no attention to relationship management with the nursing students on clinical placement who are invariably unfamiliar with the hospital setting. The study further revealed that the inability of the hospital management to specify when, how, where and what should be the commitment of the students for clinical experience made the students vulnerable to some individual behavioural issues peculiar to some preceptors and other hospital staff in the setting.

In sub-Saharan Africa involving Nigeria, nursing students going for clinical experience have over the years been subjected to complications and difficulties emanating from the hospital setting, institutional setting as well as socioeconomic factors obtainable in the environment. The challenges mostly peculiar to the students of nursing participating in clinical experience have hovered around the management, supervision, infrastructures, facilities and coordination of the activities involved in the program between the institution of higher learning and clinical facilities receiving nursing students on clinical placement.^21^^,^^22^ Most clinical facilities are ill-equipped resulting to the inability of the nursing students to have the experiential knowledge of practicing the theoretical learning in the classroom with appropriate equipment and facilities at the clinical facility settings. In some cases, some of the trusted staff of the clinical facilities mentoring the nursing students on clinical placement lacked the required skills and capacity to manage and supervise the nursing students. 

As part of the effort to develop a sustainable public health facilities and services to reach the rural population, the World Health Organization recommended students exposure to the rural health facilities in order to arouse their interest in working in the rural clinical facilities. This is to ensure adequate curative and preventive health services to the rural population especially in the developing nations where majority of the population are still located in the rural settings. However, among the sub-Sahara African nations and other developing nations, the clinical facilities in the rural areas have failed to maintain a synergy with institutions of higher learning where the nursing students and other medical students are trained, in sustaining rural rotation (RR) program among the nursing students on clinical placement. For instance, during the pilot study, this study discovered that numerous public clinic facilities have no arrangement for clinical placement for nursing students. In any case, the nursing students on clinical placement have been unconsciously restricted in the urban settings where they are carried away by the urban lifestyles and facilities that discourage the idea of working in rural clinical facilities. In the special case of the rural clinic facilities in southeast Nigeria, the infrastructures and human resources are relatively scarce and complicated owing to the long term neglect by the government.^23^^,^^24^ Owing to the three tier government structure and the developmental stage obtainable in sub-Saharan Africa such as in southeast Nigeria, public health facilities, which are mainly the destination of the nursing students on clinical placement are in dilapidated conditions and lacks the capacity to fulfil the purposes of clinical experience for the nursing students.^25^^,^^26^ The public clinical facilities in rural southeast Nigeria are characterized by absenteeism of the health workers, obsolete medical equipment, poor nurse-to-patient ratio, poor doctor-patient ratio, corruption among the health staff and more.^27^^,^^28^

The condition of the public clinic facilities in the rural communities in southeast Nigeria in view of their pertinence to effective clinical experience among the nursing students is a challenge to sustainable public health and rural development owing to the gap such situation generates in sustainable development chain analysis. For instance, the rural-urban and international migrations of the health workers experienced in Nigerian health sector today is largely connected to poor health infrastructures and welfare of the health workers, which originates from the poor management in public health facilities and institution as well as government negligence of the rural clinic facilities.^29^^,^^30^^,^^31^ For instance, the study by Chuke *et a*l. showed the truancy of the health workers in the rural clinical facilities mainly because the staff lived in the urban communities and preferred working in such vicinity than the rural communities. Equally, the studies by Yakubu *et al*.^32^and Adebayo and Akinyemi^33^ revealed that poor satisfaction among health workers in the public clinical facilities, poor remuneration, poor work environment, and insecurity especially in the rural areas as well as lack of opportunity for career development triggered the intension of health workers of emigrating to developed nations. According to the Organisation for Economic Co-operation and Development, between 2008 and 2021 United Kingdom alone received 36467 migrating medical doctors from Nigeria; between 2002 and 2021, 60 729 nurses migrated from Nigeria to the United Kingdom and this has continued to increase in other areas of medical professions.A substantive number of higher institutions of learning do send their nursing students on clinical placement in the rural clinical facilities in southeast Nigeria, owing to the fact that a significant percentage (approximately 57%) of the population live in the rural areas and these institutions equally are mostly located in-between the urban communities and the rural communities.^34^ This by implication creates the opportunity for the rural rotation (RR) program as recommended by the World Health Organization as well as given these students some feels with the rural clinic facilities. However, the missed opportunity here, which is the poor management of clinical experience among the nursing students in the rural clinical facilities and unpleasant experiences jeopardise the overall prospect of sustainable rural health in southeast Nigeria.

Clinical experience among the nursing students in places such as sub-Saharan Africa and southeast Nigeria in particular has attracted research attention of a number of scholars but in different dimensions and areas of the region. Some scholars in sub-Saharan Africa have researched on clinical placement among the nursing students in the areas of socioeconomic and personal factors affecting the nursing students on clinical placement,^35^^-^^37^ poor clinical facilities, management crises at the clinical facilities and other institutional factors affecting clinical placement among the nursing students,^38^^,^^39^ however, to the best knowledge of this study, there is yet to be a study specifically focusing on the effective clinical placement among the nursing students in the rural clinical facilities especially in southeast Nigeria rural communities. Even though some surface arguments tend to be projecting the unseen problems of poor-quality healthcare, incompetence among the health workers and some other crises, empirical evidence is lacking to substantiate the challenges and the related factors as well as to inform social and health policies suitable in dealing with the problems. The absence of empirical substantiation of these arguments has created unseen but felt gap in literature especially from the sub-Saharan Africa in the ongoing discuss on clinical experience among nursing students and sustainable rural health. This in essence warrants the present study, which aimed to fill the observed gap in literature. As such, the study is designed to answer the following research questions: (i) What are the factors affecting effective clinical experience among the nursing students in rural clinic facilities in southeast Nigeria?, (ii) What are the predicting factors to effective clinical experience among the nursing students in rural clinic facilities in southeast Nigeria?, and (iii) What are the predictors of willingness to work in rural clinic facilities if offered employment in the future among the nursing students on clinical experience in rural clinic facilities in southeast Nigeria?

## Methods

The study took place in southeast Nigerian rural communities, focusing on the community health centers and general hospitals with formal arrangement for clinical placement for nursing students from different institutions of higher learning. Due to the nature of the rural public clinic facilities in southeast Nigeria, which are not consistent in terms of capacity and functionality classifications, the study resorted to pilot study for initial classifications and development of sampling frame. From the pilot study, 92 public health centers and general hospitals were classified as active with formal arrangements for clinical placement for the nursing students from different institutions of higher learning. Clinical posting to public health facilities in the rural southeast Nigeria are limited to community health centers and the general hospitals across the local government areas with proximity to government approved higher institutions such as the federal universities, state universities, government and private universities, school of health and nursing. With the increasing insecurity in southeast Nigeria in the recent times such as mass kidnapping of commuters and other road users, the clinical posting of the nursing students has been limited to the rural communities with some level of security guarantee owing to the fact that the students do move in mass to their designated place of clinical placement. As such, the pilot study helped the researchers to group and classifies the community health centers and general hospitals in the rural locations, which are currently maintaining a formal arrangement with the higher institutions of learning for posting of nursing students on clinical experience. Also, due to the slanting of the posting towards urban health centers, the study carefully separated and classified rural communities with health centers and general hospitals engaging in clinical placement.

From the confirmed 92 rural health centers and general hospitals currently maintaining a formal arrangement with the higher institutions of learning for posting of nursing students on clinical experience, the study randomly selected 48 community health centers and general hospitals. From the 48 health facilities randomly selected for the study, quota sampling technique was applied to select 528 respondents. The study followed the season of posting and the number of institutions posting nursing students to different general hospitals and health centers included in the study. In view of the fact that virtually all the health facilities selected for the study do entertain posting of nursing students from at least two institutions of higher learning with at least three batches of posting in three seasons, the study selected 11 respondents from each of the 48 selected health facilities maintaining inclusive and exclusive criteria.

The inclusive criteria for the students to participate in the study were their coming to the facilities for the first time and not being included in this study in another facility at this period. This was because, some institutions preferred sending a set of students to more than one facility at different times in different locations in the region especially the host state within the same calendar year. The study purposely selected at least 3 students from each batch of placement from the health facilities that receives at least three batches of placement from different institutions, and equally selected at least 5 respondents from the facilities that received at least two batches of placement from different institutions of higher learning. In total, the study selected 528 respondents for the administration of research instrument. 

The study instrument was developed in nominal and ordinal scales, but was grouped into socio-demographic information of the respondents, personal factors affecting effective clinical experience, socioeconomic factors affecting effective clinical experience and institutional factors affecting effective clinical experience among the nursing students in the rural health facilities. The research instrument was administered to the respondents between November 2022 and June 2023 to accommodate the posting schedules of the different institutions involved in the clinical placement of the nursing students in the rural health facilities in order to meet the targeted population for the study. The research instrument was shared during the day in view of the fact that each group that was posted to each facility was scheduled to maintain morning, afternoon and night shifts. With the help of research assistants, the study shared 528 questionnaires to the selected respondents and these were carefully done to control bias and misplacement of the questionnaire instrument. The collected data were analyzed using descriptive and inferential statistics with the aid of SPSS version 23. At the first stage of the analysis, the collected data were presented in percentages (%) to show the socio-demographic information of the respondents, followed by the percentage (%) analysis of the substantive issues to the study such as personal and institutional factors affecting effective clinical experience among the students. The next stage of the analysis to answer the question on the predicting factors to effective clinical experience was carried out with the aid of Linear regression, which enabled the study to explore the relationship of the key variables on this with more focus on the direction of the relationship between the selected variables.

## Results

Table1 showed the socio-demographic information of the respondents. According to the table, majority of the respondents (58.3%) are females, while 41.7% are males. Majority of the respondents (46.8%) are in the age categories of 29years and above, 33.1% are in the age category of 23-28years, while 20.1% are in the age category of 17-22years. On the distribution of the marital status of the respondents, majority of the respondents (40.2%) are married, 40% are single, 13.3% are separated, while about six percent of the respondents are divorced. Among the students, majority (39.8%) are in their 4^th^ year of study, 26.7% are in their third year of study, 19.9% are in their 5^th^ year of study, while 13.6% are in their 2^nd^ year of study. Majority of the respondents (40%) source their school fees through the family, 26.5% depended on their personal income, 26.5% of the respondents are on scholarship, while about six percent depended on appeal for support. According to the socioeconomic capacities of their parents and guardians, majority of the respondents (46.4%) indicated that the monthly income of their parents/guardians is 101.000 and above; 33.3% indicated that their parents/guardians monthly income is between 71 000 and 100 000, 13.4% indicated that their parents/guardians monthly income is between 41 000 and 70 000, while about six percent indicated that their parents/guardians monthly income is between N10,000 and 40,000 Naira.

**Table 1:** Socio-demographic information of the respondents

Variables		*N*	Percentage
Respondents' gender	Males	220	41.7
	Females	308	58.3
Respondents' age	17-22	106	20.1
	23-28	175	33.1
	29and above	247	46.8
Marital status	Single	211	40.0
	Married	212	40.2
	Separated	70	13.3
	Divorced	35	6.6
Study year	2^nd^ year	72	13.6
	3^rd^ year	141	26.7
	4^th^year	210	39.8
	5^th^ year	105	19.9
Respondents' source of school fees	Appeal for support	36	6.8
	Personal income	141	26.7
	Family	211	40.0
	Scholarship	140	26.5
Parents/guardian monthly income	10 000 - 40 000	36	6.8
	41 000 - 70 000	71	13.4
	71 000 - 100 000	176	33.3
	101 000 and above	245	46.4
Total		528	100.0%

Table2 showed the distribution of the respondents on the other substantive issues to the study. According to the table, majority of the students (73.1%) indicated that they attended clinical experience regularly as scheduled, majority of the respondents (59.8%) indicated that they did all the clinical experience assignments given to them during their clinical placement while, majority of the respondents (93.4%) indicated that they partially made use of the equipment in the hospital’s laboratory for clinical practice on their own. Majority of the students (80.1%) partially did self-assessment of their performance during clinical experience, majority of the students (73.3%) indicated that they accepted corrections and ask your ward staff questions during clinical experience, while majority of the students (93.4%) indicated that they either did not, or partially used the nursing care procedure book during clinical experience as a guide for practice: during clinical experience. 

Majority of the respondents (60%) indicated that their school has no practical demonstration laboratory for students’ clinical practice, majority of the students (66.7%) indicated that their school lab does not have large space for all the students to observe what is being taught, while majority of the students (79.9%) indicated that their school lab does not have enough equipment that can enable many students to practice procedures. Majority of the students (79.9%) indicated that the hospitals where they are on clinical placement do not have enough equipment needed for the students on each shift of practice, majority of the students (59.9%) indicated that student/client ratio in each ward during clinical experience periods was not enough for students' practice under supervision, while majority of the students (73.3%) indicated that their school do not have a library with current nursing texts for references.

**Table 2:** Substantive Issues to the Study

	*n*		%
You attended the clinical experience regularly as scheduled	Not at all	36	6.8
	Rarely	106	20.1
	Sometimes	281	53.2
	Often times	105	19.9
You did all the clinical experience assignments/tests given to you during your posting (preparing patient’s room, help patient with meal and bathing, screen for health abnormality, etc.)	Not at all	71	13.4
	Rarely	141	26.7
	Sometimes	176	33.3
	Often times	140	26.5
You made use of the equipment in the hospital laboratory for clinical practice on your own	Rarely	212	40.2
	Sometimes	281	53.2
	Often times	35	6.6
You did self-assessment of your performance during clinical experience	Not at all	35	6.6
	Rarely	247	46.8
	Sometimes	176	33.3
	Often times	70	13.3
You accepted corrections and ask your ward staff questions during clinical experience	Not at all	36	6.8
	Rarely	105	19.9
	Sometimes	211	40.0
	Often times	176	33.3
You used the nursing care procedure book during clinical experience as a guide for practice: during clinical experience	Not at all	70	13.3
	Rarely	247	46.8
	Sometimes	176	33.3
	Often times	35	6.6
Your school has practical demonstration laboratory for students’ clinical practice	S-D	71	13.4
	Disagree	246	46.6
	Agree	140	26.5
	S-A	71	13.4
Your school lab has large space for all the students to observe what is being taught	S-D	106	20.1
	Disagree	246	46.6
	Agree	140	26.5
	S-A	36	6.8
Your school lab has enough equipment that can enable many students to practice procedures	S-D	140	26.5
	Disagree	282	53.4
	Agree	106	20.1
The hospital has enough equipment needed for the students on each shift of practice	S-D	106	20.1
	Disagree	316	59.8
	Agree	106	20.1
Student/client ratio in each ward during clinical periods is enough for students' practice under supervision	S-D	70	13.3
	Disagree	246	46.6
	Agree	212	40.2
Your school has a library with current nursing texts for references	S-D	105	19.9
	Disagree	282	53.4
	Agree	141	26.7
Total		528	100.0%

The model predicted the effectiveness of clinical experience among the nursing students in rural health facilities in southeast Nigeria *R* ^*2*^ *=.763, F (170.553).* According to the model several factors included in the model significantly contributed in the explanation of effective clinical experience among the nursing students on clinical placement in rural health facilities; these factors included gender of the respondents, study years of the respondents, source of their school fees, parents/guardians’ monthly income, institutional factors (1&2) as well as the willingness to serve in the rural health facilities after graduation. Factors such as gender, source of school fees, monthly income of the parents/guardians, personal factors and willingness to serve in the rural health facilities after graduation contributed in the positive prediction of the likelihood of effective clinical experience among the nursing students on clinical placement in the rural health facilities. 

Meanwhile, factors such as study years, institutional factors (1&2) all negatively predicted the likelihood of effective clinical experience among the nursing students on clinical placement in the rural health facilities. Gender in this context is significant in the model owing to the number of women involved in the study as nursing career in this part of the world is still seen as women career; this seems to play out in the extent of commitment that can come from the female folks participating in the career. Also, following the coding of the source of school fees of the students in the positive calibration, the presence and position of this factor in the model showed that the more comfortable the students are in terms of school fees sponsorship, the more likely they are going to be effective in the clinical experience; this also applies to the monthly income of their sponsors. Nonetheless, personal factors as positively significant in the model showed the passion for the career among the students as impacting on the effectiveness of their clinical experience. (Table 3)

**Table 3:** Coefficients of effective clinical experience

	Unstandardized Coefficients		Standardized Coefficients			95.0% Confidence Interval for B	
Model	B	Std. Error	Beta	T	Sig.	Lower Bound	Upper Bound
(Constant)	-0.439	0.228		-1.925	0.055	-0.887	0.009
Respondents' gender	0.458	0.054***	0.267	8.421	<0.001	0.351	0.565
Respondents' age	0.039	0.063	0.036	0.628	0.530	-0.084	0.163
Marital status	-0.004	0.025	-0.004	-0.172	0.864	-0.053	0.045
Study year	-0.290	0.040***	-0.321	-7.304	<0.001	-0.368	-0.212
Respondents' source of school fees	0.419	0.034***	0.435	12.299	<0.001	0.352	0.486
Parents/guardian monthly income	0.582	0.058***	0.621	10.050	<0.001	0.468	0.695
Personal factors among the students	0.115	0.051*	0.109	2.271	0.024	0.016	0.215
Institutional factor^1^ (Institution of training)	-0.263	0.057***	-0.271	-4.634	<0.001	-0.375	-0.152
Institutional factor^2^ (rural clinic facility)	-0.149	0.070*	-0.110	-2.123	<0.001	-0.287	-0.011
Willingness to working in rural health facilities after graduation	0.299	0.025***	0.329	12.157	<0.001	0.251	0.348

a. Dependent Variable: Effective Clinical Experience

df 10, *p<0.05, ** p<0.01, *** p<0.001, R = 0.876 (76.7) R ^*2*^ *=.763 (58.2), F (170.553)*

The above model predicted the effectiveness of clinical experience among the nursing students in rural health facilities in southeast Nigeria *R* ^*2*^ *=.763, F (170.553).* According to the model several factors included in the model significantly contributed in the explanation of effective clinical experience among the nursing students on clinical placement in rural health facilities; these factors included gender of the respondents, study years of the respondents, source of their school fees, parents/guardians monthly income, institutional factors (1&2) as well as the willingness to serve in the rural health facilities after graduation. Factors such as gender, source of school fees, monthly income of the parents/guardians, personal factors and willingness to serve in the rural health facilities after graduation contributed in the positive prediction of the likelihood of effective clinical experience among the nursing students on clinical placement in the rural health facilities. 

Meanwhile, factors such as study years, institutional factors (1&2) all negatively predicted the likelihood of effective clinical experience among the nursing students on clinical placement in the rural health facilities. Gender in this context is significant in the model owing to the number of women involved in the study as nursing career in this part of the world is still seen as women career; this seems to play out in the extent of commitment that can come from the female folks participating in the career. Also, following the coding of the source of school fees of the students in the positive calibration, the presence and position of this factor in the model showed that the more comfortable the students are in terms of school fees sponsorship, the more likely they are going to be effective in the clinical experience; this also applies to the monthly income of their sponsors. Nonetheless, personal factors as positively significant in the model showed the passion for the career among the students as impacting on the effectiveness of their clinical experience. (Table 3)

From the findings, 19.9% of the respondents strongly disagreed that they will be working in rural health facilities after graduation, 40.3% disagreed, 26.5% agreed, while 13.3% strongly agreed that they will be working in the rural health facilities after graduation.The model predicted willingness to work in rural health facilities after graduation among the nursing students on clinical placement in the rural health facilities in southeast Nigeria R^2^=0.523 (27.4), F (56.634). A number of factors contributed in the model in predicting the likelihood of working in the rural health facilities after graduation among the nursing students on clinical placement in the rural health facilities in southeast Nigeria; among the factors are, gender, age, respondents’ source of school fees, parents/guardians monthly income, personal factors among the students, institutional factors (1&2) and effective clinical experience. However, only parents/guardians monthly income and effective clinical experience contributed to the explanatory power of the model in the positive dimension. The willingness to serve in the rural health facilities among the upcoming nurses is one of the unseen factors affecting the sustainability of rural health workers especially the nurses who make up the 60% of the rural health workers in the southeast Nigeria. And this factor in its respect is dependent on other factors, which the present findings have revealed. Women health workers are more likely to prefer urban setting for their careers than their male counterparts, this is confirmed in the findings of the model as majority of the participants in this study are females. (Table 4)

**Table 4:** Coefficients of Willingness to work in rural health facilities after graduation

	Unstandardized Coefficients			Standardized Coefficients				95.0% Confidence Interval for B
Model	B	Std. Error	Beta	T	Sig.	Lower Bound	Upper Bound
(Constant)	3.482	0.326		10.673	<0.001	20.841	40.122
Respondents' gender	-0.388***	0.090	-0.206	-4.332	<0.001	-0.565	-0.212
Respondents' age	-0.542***	0.096	-0.446	-5.659	<0.001	-0.730	-0.354
Marital status	-0.020	0.039	-0.019	-.513	0.608	-0.097	0.057
Study year	0.008	0.066	0.008	.125	0.901	-0.121	0.137
Respondents' source of school fees	-0.507***	0.057	-0.478	-8.925	<0.001	-0.619	-0.396
Parents/guardian monthly income	0.357***	0.098	0.346	3.624	<0.001	0.163	0.550
Personal factors among the students	-0.267**	0.079	-0.229	-3.358	0.001	-0.423	-0.111
Institutional factor^1^ (Institution of training)	-0.271*	0.091	-0.254	-2.996	<0.001	-0.449	-0.093
Institutional factor^2^ (rural clinic facility)	-0.196	0.111	-0.132	-1.768	<0.001	-0.022	0.413
Effective Clinical Experience	0.743***	0.061	0.675	12.157	<0.001	0.623	0.863
				

a. Dependent Variable: Willingness to work in rural health facilities after graduation

df 10, *p<0.05, ** p<0.01, *** p<0.000, R = 0.723 (52.3) R ^*2*^ *=0.523 (27.4), F (56.634)*

## Discussion

In this study, it was specified as a research objective, to understand the factors affecting effective clinical experience among the nursing students in the rural clinic facilities with focus on the personal, socioeconomic and institutional factors. Although these factors have been explored by other researchers, the uniqueness of the present approach is found in the context of the study, which is southeast Nigeria, elements of rurality and rural health sustainability. From the findings of the study, socioeconomic, personal and institutional factors appeared to have sway over effective clinical experience rather negatively. Among the nursing students studied, the institutional factors (here which were categorized as clinic facilities as institution and institutions of higher learning), have affected the potentials of the nursing students for effective clinical experience. This challenge is anchored on the failed responsibilities of these institutions to provide the necessary basic equipment and facilities to aid the clinical experience for the students on clinical placement. This is reflected on the elements of responsibilities by these institutions, which were explored in the study; for instance, majority of the respondents (60%) indicated that their school has no practical demonstration laboratory for students’ clinical practice, majority of the students (66.7%) indicated that their school lab do not have large space for all the students to observe what is being taught, while majority of the students (79.9%) indicated that their school lab do not have enough equipment that can enable many students to practice procedures. Majority of the students (79.9%) indicated that the hospitals where they are on clinical placement do not have enough equipment needed for the students on each shift of practice, majority of the students (59.9%) indicated that student/client ratio in each ward during clinical experience periods was not enough for students' practice under supervision, while majority of the students (73.3%) indicated that their school do not have a library with current nursing texts for references. basically, these two institutions as categorized in this study are the cardinal points to achieve effective clinical experience among the students through the expected provision of the basic facilities and synergy aimed at cultivating discipline in the students at the cause of the clinical placement program. The availability of the basic equipment and facilities at this stage of the students’ training are necessary to give them familiarities they needed in the nursing career for future operation. From the studies of other scholars, the inability of nurses to use medical equipment properly have been found as connected to inefficiency among some nurses resulting to preventable deaths of patients and other preventable complications.^40^^,^^41^

The clinical experience for the nursing students is usually the much opportunity they have to interact with the atmosphere of managing health situations and health facilities especially the emergency and specialized equipment and approaches such as medications, first aid supplies, air way management, patient transport, personal protective equipment, injection and IV supplies, etc.; missing the opportunity of learning to handle these equipment and situations at this stage is an alteration of the training procedure bound to cause crises for the students and their future place of career. This applies to self-assessment and using nursing care procedure book, which are practical procedures for adapting into the overall nursing career and health management. From the findings of this study, majority of the respondents (93.4%) indicated that they did not or partially made use of the equipment in the hospital’s laboratory for clinical practice on their own; 80.1% partially did self-assessment of their performance during clinical experience, while 93.4% indicated that they either did not, or partially used the nursing care procedure book during clinical experience as a guide for practice. In comparison to the findings of other studies, inability of the nursing students on clinical experience to do self-examination has been discovered as responsible for practice complication and disorderliness in the latter stage of their career. Nursing staffs who applied self-assessment during clinical experience have been found more efficient than those who did not during clinical experience.^42^^-^^46^ Equally, some studies have reported the relationship between observing and using nursing care procedure book during clinical experience and efficiency of nursing practice in the latter stage of the profession.^47^^,^^48^ In essence, the inability of the students to maintain self-assessment and the use of nursing care procedure book due to poor supervision and poor facilities are potential challenges in the future practice of the profession for the students involved.

In a correlation model to check for the predicting factors to effective clinical experience among the nursing students serving in rural health facilities, this variable (effective clinical experience) is predicted by such factors as gender, source of school fees, parents’ source of income, institutional factors (1&2) and willingness to serve in the rural health facilities after graduation. As evidence of many females involving the study, being a female according to the model, is much likely to positively affect effective clinical experience in the rural clinic facilities. This finding support other related studies in this area, which have shown that women are more likely to do well in the nursing profession than their male counterparts.^49^^,^^50^ In the issues of institutional factors (the clinic facilities and the facilities in the institutions of higher learning), which appeared with negative signs in the model, the hospital facilities and school facilities appeared to be hindering effective clinical experience among the students on clinical placement in the rural health facilities; this is evidence in the number of factors connected to the health and school facilities such as provision of equipment and materials for successfully undergoing clinical experience by the students. In further clarification of the negative significance of the institutional factors on the model,60% of the students indicated that their school has no practical demonstration laboratory for students’ clinical practice, majority of the students (66.7%) indicated that their school lab do not have large space for all the students to observe what is being taught, while majority of the students (79.9%) indicated that their school lab do not have enough equipment that can enable many students to practice procedures. Majority of the students (79.9%) indicated that the hospitals where they are on clinical placement do not have enough equipment needed for the students on each shift of practice, majority of the students (59.9%) indicated that student/client ratio in each ward during clinical experience periods was not enough for students' practice under supervision, while majority of the students (73.3%) indicated that their school do not have a library with current nursing texts for references. The finding corroborates with the findings of other studies such as the ones by Akyüzand Ergöl;^51^ Jacob *et al*;^52^ Hakim;^53^ Fego *et al.*^54^ who concluded from their studies that institutional facilities are the heaviest challenges to clinical experience by the nursing students and other medical students in the developing nations.

The possibility of serving in rural health facilities by the nursing students after graduation was examined using statistical model; from the model, certain factors predicted the probability of nursing students having their clinical experience in the rural clinic facilities, showing willingness to return as staffs in such and similar facilities in the future if given employment. From the findings of the model, the higher the age of the students, the more likelihood that they will not be interested in serving in the rural clinic facilities if given employment as a nurse in the future. Other factors such as gender, age, socioeconomic status and the individual preferences appeared as negatively predicting the probability of the nursing students on clinical placement in the rural clinic facilities returning in the future if offered employment. Related studies on brain drain issues in Nigeria and other developing nations have indicated the above mentioned factors,^55^^-^^59^ but the present study deepened the knowledge by focusing on the rural clinic facilities and the future nurses. Sustainability of rural health in the developing nations such as in southeast Nigeria is much dependent on the process of training the health workers in the institutions of higher learning, which includes familiarity with the rural clinic facilities. This could be realized through the clinical placement among the nursing students, which technically condition them to go for practical experience in different clinical facilities during the cause of their training. This is captured by the recommended World Health Organization’s rural rotation (RR) program, which aims at exposing the medical (nursing) students to the rural health facilities.^60^^,^^61^

Health management in the rural southeast Nigeria is unique in its own appearance compared to other regions and conventional expectations. This is visible in the patients/nurses ratio in the region, which is one of the poorest in the world (1:160 compared to the WHO standard of 1:5).^62^^-^^64^ In rural southeast Nigeria, much of the health management burdens are on the nurses owing to the shortages of medical doctors and other specialists. This by implication has placed much demand on the recruitment and training of nurses in government and private institutions of higher learning. Although the problem of paramedics with little or poor training who now become cheap labor (nurses and more) for most private hospitals in the rural communities in the region has dominated the atmosphere of rural health, the few public health centers and general hospitals in the rural communities still operate to manage the rural population. And, the bulk of the human resources to maintain these facilities in the rural communities come from the students trained by the government and private institutions due to the extant employment laws that the government health facilities cannot employ any staff with less than certificates obtained from government approved institutions. As such, the nursing students who are attending the clinical experiences in these health facilities in most cases become the next generation to be employed in the health system involving the rural health facilities. As a concern to this study, the way and manner these nurses are trained determines the future fate of the rural health facilities management to some extent.

The implication of the findings of this study, which showed the factors surrounding effective clinical experience in the rural health facilities in the southeast Nigeria, is the need for urgent attention to the factors indirectly fueling discouragement of the nurses and other health workers from engaging with the rural health facilities in southeast Nigeria. Rural health in the developing regions such as southeast Nigeria is gradually becoming obsolete owing to several factors including the ones revealed in this study. According to the United Nations Sustainable Development Goal3, there is need for health and wellbeing for all across urban and rural community’s world over of which the situation in southeast Nigeria rural communities negates as many rural clinic facilities are virtually empty due to the chronic problem of rural-urban migration of the nurses and other health workers. More importantly as this study has revealed, the problem of desertion of the rural clinic facilities by the upcoming nurses and perhaps, other health workers are connected to institutional factors directly anchored on the nature of the rural clinic facilities and the institutions of higher learning. With the current challenges as the students are encountering, the system is gradually creating a repulsive atmosphere against the future potential rural health workers in this region.
